# 'Communicate to vaccinate' (COMMVAC). building evidence for improving communication about childhood vaccinations in low- and middle-income countries: protocol for a programme of research

**DOI:** 10.1186/1748-5908-6-125

**Published:** 2011-12-02

**Authors:** Simon Lewin, Sophie Hill, Leyla H Abdullahi, Sara Bensaude de Castro Freire, Xavier Bosch-Capblanch, Claire Glenton, Gregory D Hussey, Catherine M Jones, Jessica Kaufman, Vivian Lin, Hassan Mahomed, Linda Rhoda, Priscilla Robinson, Zainab Waggie, Natalie Willis, Charles S Wiysonge

**Affiliations:** 1 Norwegian Knowledge Centre for the Health Services, PO Box 7004 St. Olavs plass N-0130 Oslo, Norway; 2 Health Systems Research Unit, Medical Research Council of South Africa, Cape Town, South Africa; 3 Centre for Health Communication and Participation, Australian Institute for Primary Care & Ageing, La Trobe University, Bundoora, Victoria 3086, Australia; 4 Vaccines for Africa Initiative, Institute of Infectious Disease and Molecular Medicine, University of Cape Town, Anzio Road, Observatory 7925, South Africa; 5 School of Child and Adolescent Health, University of Cape Town, Anzio Road, Observatory 7925, South Africa; 6 International Union for Health Promotion and Education (IUHPE/UIPES), 42 Boulevard de la Liberation, 93203 Saint Denis Cedex, France; 7 Swiss Tropical and Public Health Institute, University of Basel. Socinstrasse 57, 4051 Basel, Switzerland; 8 School of Public Health, La Trobe University, 215 Franklin Street, Melbourne, Victoria 3000, Australia; 9 South African Tuberculosis Vaccine Initiative, Institute of Infectious Disease and Molecular Medicine, University of Cape Town, Anzio Road, Observatory 7925, South Africa

## Abstract

**Background:**

Effective provider-parent communication can improve childhood vaccination uptake and strengthen immunisation services in low- and middle-income countries (LMICs). Building capacity to improve communication strategies has been neglected. Rigorous research exists but is not readily found or applicable to LMICs, making it difficult for policy makers to use it to inform vaccination policies and practice.

The aim of this project is to build research knowledge and capacity to use evidence-based strategies for improving communication about childhood vaccinations with parents and communities in LMICs.

**Methods and design:**

This project is a mixed methods study with six sub-studies. In sub-study one, we will develop a systematic map of provider-parent communication interventions for childhood vaccinations by screening and extracting data from relevant literature. This map will inform sub-study two, in which we will develop a taxonomy of interventions to improve provider-parent communication around childhood vaccination. In sub-study three, the taxonomy will be populated with trial citations to create an evidence map, which will also identify how evidence is linked to communication barriers regarding vaccination.

In the project's fourth sub-study, we will present the interventions map, taxonomy, and evidence map to international stakeholders to identify high-priority topics for systematic reviews of interventions to improve parent-provider communication for childhood vaccination. We will produce systematic reviews of the effects of high-priority interventions in the fifth sub-study. In the sixth and final sub-study of the project, evidence from the systematic reviews will be translated into accessible formats and messages for dissemination to LMICs.

**Discussion:**

This project combines evidence mapping, conceptual and taxonomy development, priority setting, systematic reviews, and knowledge transfer. It will build and share concepts, terms, evidence, and resources to aid the development of communication strategies for effective vaccination programmes in LMICs.

## Background

Vaccination is one of the most cost-effective public health interventions to significantly reduce childhood mortality and morbidity [1]. It has widespread public health benefits and is viewed as a public good [2,3]. Strategies that increase vaccination uptake involve 'supply-side' components, such as availability of effective vaccines, adequate health systems to support their delivery and health personnel to administer the vaccines [4]; and 'demand-side' components, such as household and individual determinants (*e.g*., building the knowledge and agency of individuals to utilise such programmes to their benefit). Particularly relevant examples of demand-side components are those addressing barriers to vaccination associated with parental knowledge, understanding, attitudes, beliefs, and behaviours [5-10], such as strategies to remind parents to get their children vaccinated [11]. In research and policy, more attention has focussed on supply-side approaches to the neglect of the demand-side [7,12].

Despite increases in vaccination rates in many countries in the last decade, great inequities exist between and within countries. Besides this, more than 24 million children still do not have access to basic immunisation services [7,13]. Increasing vaccination rates among disadvantaged sectors of a community remains a challenge, for practical, social, and socio-political reasons [14-16].

## The project

The Communicate to Vaccinate (COMMVAC) project is a part of the Global Health and Vaccination Research (GLOBVAC) programme, funded by the Research Council of Norway. The main aim of the COMMVAC project is to build research knowledge and capacity to use evidence-based strategies for improving communication about childhood vaccinations with parents and communities in low- and middle-income countries (LMICs).

## Communication for vaccination

Effective communication has the potential to improve childhood vaccination uptake, address partial immunisation, further strengthen routine immunisation services, and increase the use of new and underused vaccines in LMICs.

A communication intervention can be defined as a purposeful, structured, repeatable, and adaptable strategy [17] to inform and influence individuals, communities, or health services and systems. Communication interventions may affect consumers' participation in health initiatives, disease prevention and promotion programmes, policy making, service management, and research. Such interventions may be implemented through diverse forms of media. For the sake of brevity, we use the phrase 'communication interventions' to refer to a wide variety of interventions to inform, educate, communicate with, involve, and support people in the management of their health [18].

A range of such interventions may be utilised in vaccine programmes. These may operate at individual, community, or societal levels, and target people in their role as parents or community members. In the context of childhood vaccination policy and programmes, communication strategies may encompass a variety of interventions, such as: public information campaigns [19]; education strategies that are tailored to local cultures [20] or accessible to those with low health literacy [21]; addressing missed vaccinations with reminder systems [22]; giving parents the information to assess and manage side effects [23]; and involving community members in planning and evaluating programmes [24].

## Finding rigorous evidence of the effects of communication interventions

Evidence is available from individual studies on the effects of a range of communication interventions, but there are few systematic reviews that have critically appraised and summarised existing research. Furthermore, LMIC programme managers and policy makers who try to seek guidance for developing new approaches to addressing barriers to vaccination uptake may find that the available information does not appear to be applicable to LMICs because these studies were conducted in very different social contexts and health systems settings. Systematic identification and review of the range of interventions used in practice is therefore needed urgently. This should describe the scope of interventions available and provide information on how they operate, how best to deliver them, and their effects across different settings. It should also examine the applicability of these findings across different contexts.

This project will draw from and build upon existing specialised knowledge resources for describing, categorising, and evaluating interventions to improve communication with people about their health and healthcare [17,25]. The terminology has been developed within the health communication domain to create a shared language so that knowledge resources-- including concepts, practices, interventions, and evidence--can be universally understood and used to promote improvements in health outcomes.

## Methods and design

The COMMVAC project is an international collaboration, with project partners in Norway, Australia, South Africa, France, and Switzerland. The project's principal investigator is located in Oslo, Norway (SL).

**Figure 1:**
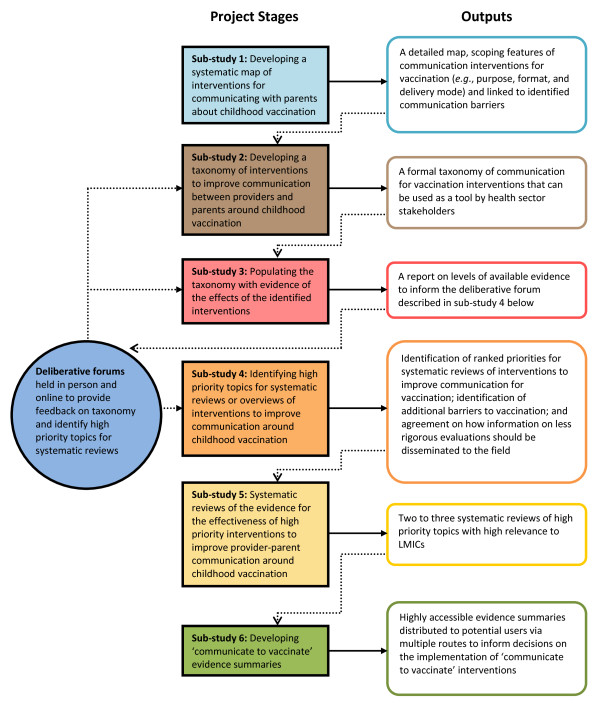
**Flow chart of the stages of the COMMVAC project**.

The project is divided into six stages, or sub-studies. Each sub-study will build upon the outputs of the previous sub-studies (see Figure 1).

All of the data used in this project will be obtained from published studies or from public deliberative forums and therefore do not raise significant ethical issues. Ethical approval will be sought in Norway for the deliberative forums because this will involve documenting the discussions of key stakeholders and summarising these for distribution in the public domain.

A project advisory group will be established to advise on the direction and implementation of the project; make suggestions regarding where to seek specialist advice; provide input on the interpretation of findings and the development of recommendations; and advise on, and promote dissemination of the project findings.

## Sub-study one: Developing a systematic map of interventions for communicating with parents about childhood vaccination

### Rationale

Globally, a wide range of interventions that involve communication to improve vaccination has been developed and evaluated, but this disparate evidence is not available in an organised manner that can inform decision making. It is also not clear to what extent available interventions address barriers to vaccination identified by or relevant to stakeholders, including consumers and programme managers. Systematically mapping the evidence provides an explicit means to identify the scope of available interventions and the quality of evidence on their effectiveness, assess where evidence 'gaps' exist, and determine options for future programme planning [26].

### Methods

Systematic maps draw on the same rigorous and transparent methods used for systematic reviews of effects, but focus on documenting and describing the range of interventions available rather than their effects [27,28]. We will search multiple databases for literature describing or evaluating communication interventions related to childhood vaccination, selecting on key criteria of communication intervention (defined comprehensively as per the Cochrane Consumers & Communication Review Group scope [25]) and childhood vaccination. The databases of primary focus will be the Cochrane Central Register of Controlled Trials (CENTRAL) and MEDLINE. For each communication intervention identified, we will extract descriptive information on the aim of the intervention, population(s) targeted, intervention content and tools used to deliver the intervention, evaluation design (where applicable), and outcomes assessed [29]. We will also link these interventions to known barriers to improved vaccination uptake [8,30] and examine relationships between intervention types and delivery context.

The construction of the interventions map will entail extracting and mapping the following data from all included studies, with particular attention paid to capturing information about interventions in a systematic and structured way [29,31]:

1. Communication barriers identified (if any)

2. Population features: population group; setting of vaccination services (*i.e.*, country and region, urban, or rural); vaccination delivery strategy, details of vaccine dose and coverage; and age of infants or children

3. Communication intervention features: intervention purpose; direction of communication [32]; parties involved; content of communication; format and delivery mode; deliverer (*i.e.*, service type or personnel); training required; setting; frequency or timing of communication; and cost

4. Outcome features: main outcomes measured (where applicable); and side effects.

Rigorous effects studies (*i.e.*, controlled trials) will be evaluated on additional characteristics using existing checklists. These data will allow us to describe where, in relation to the interventions map, robust evidence of effectiveness is available, informing later project sub-studies.

### Output

The output of sub-study one is a detailed map, scoping features of communication interventions for vaccination (*e.g*., purpose, format, and delivery mode) and linked to identified communication barriers.

## Sub-study two: Developing a taxonomy of interventions to improve communication between providers and parents around childhood vaccination

### Rationale

Despite the importance of communication to vaccine delivery and uptake, we are not aware of any comprehensive approach to organising, and therefore understanding, communication interventions related to childhood vaccination. Developing a classification system, organised into categories based on conceptual or practical similarities (a taxonomy), of communication interventions will help to: understand the relationships between different types of interventions in the field; facilitate conceptual mapping of these interventions; clarify the key purposes and features of interventions, thereby assisting with implementation and evaluation; and identify areas where evidence is strong and where there are gaps. While the intervention map (sub-study one) will provide a descriptive overview of the 'landscape' of communication for vaccination, the taxonomy will provide a relatively discrete set of types of interventions and illustrate relationships between differently described interventions.

### Methods

We will build on taxonomies already developed, including: a comprehensive listing of all interventions for communication in health, organised by direction of communication [25]; and a taxonomy of interventions directed to consumers for evidence-based prescribing [33]. We will develop the vaccination communication taxonomy by using known barriers to improving communication for vaccination as well as the studies identified in sub-study one. We will also refine the taxonomy through discussions with communication and vaccination experts and potential users from a range of settings.

### Output

The output of sub-study two is a formal taxonomy of communication for vaccination interventions that can be used as a tool by health sector stakeholders to identify gaps and research priorities, build future evidence, and clarify purposes for communication.

## Sub-study three: Populating the taxonomy with evidence of the effects of the identified interventions

### Rationale

A key step towards building a knowledge base is being able to identify, in relation to the categories of communication interventions identified in the taxonomy: where high-quality evidence of effectiveness exists; where there are potentially useful interventions but little high-quality evidence of their effects; and where interventions are being mentioned but have not been evaluated.

### Methods

The literature identified in sub-study one will be used to populate the taxonomy with citations of trials, or less rigorous evaluations where trials do not exist. We will then examine in detail each of the taxonomy categories to ascertain where rigorous effectiveness evaluations exist and where there are major knowledge gaps regarding effects. We will first consider the global evidence and then the evidence available from LMICs.

### Output

The output of sub-study three is a report on levels of available evidence to inform the deliberative forums described in sub-study four below.

## Sub-study four: Identifying high-priority topics for systematic reviews or overviews of the effect of interventions to improve communication around childhood vaccination

### Rationale

Resources to conduct high-quality systematic reviews of effects are limited. The involvement of key stakeholders in setting priorities for systematic reviews of effect will help to ensure the relevance of review outputs for future service provision. Stakeholders include government and non-government agencies, bilateral and multilateral agencies, global health initiatives, and programme personnel, as well as consumer groups.

### Methods

We will hold a series of deliberative forums for key stakeholders [34,35]. The purposes of the forums will be to discuss the outputs of the previous stages; comment on the rigour of the available evaluations of interventions and contribute practice knowledge that is missing from the research; ensure that issues of setting, culture, resources, and equity have been considered adequately; and discuss and agree on priorities for systematic reviews and further evaluations of the effect of interventions.

### Output

Outputs of sub-study four include: identification of ranked priorities for systematic reviews of interventions to improve communication for vaccination; identification of additional barriers to vaccination; and agreement on how information on less rigorous evaluations should be disseminated to the field. Feedback on the taxonomy will be incorporated into updated versions of the outputs of sub-studies two and three through an iterative process (see Figure 1).

## Sub-study five: Systematic reviews of the effect of high-priority interventions to improve provider-parent communication around childhood vaccination

### Rationale

Systematic reviews of effect will contribute to bridging evidence gaps on communication interventions for promoting vaccination uptake.

### Methods

The reviews will focus on two to three high-priority review topics identified in sub-study four, and will be conducted according to the methods recommended by the Cochrane Collaboration [36,37].

### Outputs

The outputs of sub-study five are two to three systematic reviews of the effect of interventions with high relevance to LMICs.

## Sub-study six: Developing 'communicate to vaccinate' evidence summaries

### Rationale

Policy makers and managers wanting to make well-informed decisions about how best to improve communication for vaccination need reliable, accessible, and up-to-date evidence. However, systematic reviews are generally not easily accessible to policymakers. Furthermore, it is often difficult for review users to know how confident they should be in the quality of the evidence as well as in its implications and local applicability.

### Methods

Drawing on an approach that the authors have piloted extensively [38-40], we will produce 'evidence bulletins' summarising evidence from high-priority systematic reviews identified or produced in this project. Each bulletin will include: key findings; an assessment of the confidence that can be placed in these findings [41]; and a discussion of key implementation issues, such as applicability in LMICs, equity impacts and monitoring needs. The bulletins will also summarise lessons emerging from the project. They will be disseminated through networks such as the 'Vaccines for Africa' initiative and the International Union for Health Promotion and Education (IUHPE).

### Outputs

The outputs for sub-study six are highly accessible evidence summaries distributed to potential users via multiple routes to inform decisions on the implementation of 'communicate to vaccinate' interventions.

## Discussion

There are significant knowledge gaps regarding how more effective communication between parents and healthcare providers can be used to improve childhood vaccination rates in LMICs. However, conducting new primary research in the absence of a comprehensive synthesis of existing knowledge is wasteful. We propose to synthesise and build on what is already known and evaluated, in a research design that is informed by the priorities of key stakeholders, including consumers. The COMMVAC project will not only provide useful, applicable evidence of the effectiveness of relevant interventions through systematic reviews, it will also produce a valuable map of the existing evidence landscape. This map can be used to identify gaps and prioritise future primary and secondary research.

Efforts to improve vaccination coverage in LMICs are central to meeting the Millennium Development Goal (MDG) of reducing child mortality [42]. While many of the vaccines needed to save children's lives already exist, improvements in coverage will depend in part on more effective communication between healthcare providers and the parents and caregivers of children. The COMMVAC project will add substantially to our understanding of 'communication for vaccination'--a neglected area of study. Working with key immunisation stakeholders, this project will produce high-quality evidence on effective communication strategies for vaccination in LMICs and will package this evidence in formats that are accessible to programme managers. Through these outputs, the project has the potential to improve child health and contribute to meeting the MDGs. More effective communication may also have wider impacts through improving care quality for other health services for children and adults.

## Competing interests

The authors declare that they have no competing interests.

## Authors' contributions

SL and SH led the conceptualisation and design of the project, prepared the original project proposal and obtained funding. CJ, GH, PR, VL, and XBC contributed to the original proposal. SH and JK developed this manuscript from the project proposal with input from SL, VL, NW, and CSW. All authors provided input into various aspects of the project, provided ongoing feedback and approved the final version of the manuscript.
